# Tomographic diagnosis of alveolar bone coverage impact in orthodontic planning: cross-sectional study

**DOI:** 10.1590/2177-6709.29.5.e242446.oar

**Published:** 2024-10-07

**Authors:** Katia MONTANHA-ANDRADE, Paula Paes FERREIRA, Ana Carolina Velasco Pondé DE SENA, Patricia R. CURY, Ieda M. CRUSOÉ-REBELLO

**Affiliations:** 1 Federal University of Bahia, School of Dentistry, Department of Dentistry and Health (Salvador/BA, Brazil).; 2 Federal University of Bahia, School of Dentistry, Department of Periodontics (Salvador/BA, Brazil).; 3 Federal University of Bahia, School of Dentistry, Department of Dentomaxillofacial Radiology (Salvador/BA, Brazil).

**Keywords:** Bone, Cone-beam computed tomography, Cross-sectional study, Orthodontics, Osso, Tomografia computadorizada de feixe cônico, Estudos transversais, Ortodontia

## Abstract

**Introduction:**

Alveolar bone coverage can be diagnosed through cone beam computed tomography (CBCT) and this information can prevent orthodontic tooth movement beyond the biological limit.

**Objective:**

This study evaluated the impact of the bone coverage (BC) diagnosis by CBCT in the orthodontists’ planning.

**Methods:**

One hundred fifty-nine Brazilian orthodontists suggested treatment plans for six patients at two different times, using two sequential questionnaires. The first questionnaire consisted of extra and intra-oral photographs, one panoramic radiograph; one lateral cephalometric radiograph with Steiner and Tweed analysis, and the patient chief complaint. The second questionnaire included the same presentations of cases with tomographic images and the radiologist’s report. The McNemar test assessed the difference between the first and the second treatment plans.

**Results:**

In all six cases, most participants changed the treatment plan after evaluating the CBCT images and the radiologist’s report (93.7% in case 5, 78.6% in case 4, 74.2% in case 3, 69.8% in case 6, 66% in case 2 and 61% in case 1; *p*≤0.01).

**Conclusion:**

The evaluation of bone coverage through CBCT images has a substantial impact on the orthodontic diagnosis and planning of the Brazilian orthodontists.

## RESUMO

**Introdução:**

A cobertura óssea alveolar pode ser diagnosticada por meio da tomografia computadorizada de feixe cônico (TCFC), e as informações podem evitar movimentos dentários ortodônticos para além do limite biológico.

**Objetivo:**

Avaliar o impacto do diagnóstico da cobertura óssea alveolar por TCFC no planejamento ortodôntico.

**Métodos:**

159 ortodontistas brasileiros sugeriram planos de tratamento para seis pacientes em dois tempos diferentes, utilizando dois questionários sequenciais. O primeiro questionário consistiu em fotografias intra e extrabucais, radiografias panorâmica e cefalométrica lateral com análise de Steiner e Tweed, e a queixa do paciente. O segundo incluiu as mesmas apresentações dos casos, com imagens tomográficas e o laudo do radiologista. O teste de McNemar avaliou a diferença entre o primeiro e o segundo plano de tratamento.

**Resultados:**

Em todos os seis casos, a maioria dos participantes alterou o plano de tratamento após avaliar as imagens de TCFC e o relatório do radiologista (93,7% no caso 5, 78,6% no caso 4, 74,2% no caso 3, 69,8% no caso 6, 66% no caso 2 e 61% no caso 1; *p*≤0,01).

**Conclusões:**

A avaliação da cobertura óssea por meio das imagens tomográficas possui impacto substancial no diagnóstico e planejamento ortodôntico dos ortodontistas brasileiros.

## INTRODUCTION

Radiography examinations are essential in orthodontic diagnosis and treatment planning - for example, two-dimensional (2D) imaging was used satisfactorily for many years. However, some limitations of 2D examinations make it difficult to analyze some structures, such as: overlapping images, poor sharpness, magnification, and distortion.^1^ ^-^ ^3^ The advent of cone-beam computed tomography (CBCT) allowed the evaluation of previously inaccessible areas and structures, like the buccal and lingual alveolar plates and resorptive lesions.^4^ ^-^ ^7^ CBCT is recommended to analyze root resorption, supernumerary teeth, temporomandibular joint pathology, and limits of alveolar bone.^8^ ^-^ ^10^ Orthodontists increasingly rely on three-dimensional (3D) imaging techniques such as CBCT for comprehensive treatment planning. However, the utilization of 3D imaging presents several challenges. Firstly, interpreting volumetric data requires specialized training and expertise, which may not be readily available to all orthodontists. Secondly, there are concerns regarding radiation exposure associated with CBCT scans. Additionally, the cost and accessibility of CBCT machines pose logistical challenges for some orthodontic practices.

Alveolar bone coverage is a crucial diagnostic parameter for orthodontic treatment planning, especially in cases in which small inclinations of teeth can cause irreversible damage to the periodontal tissue, and is not routinely assessed through imaging.^10^ ^-^ ^16^ One of the most relevant aspects of CBCT examinations is the evaluation of buccal and lingual bone thicknesses before orthodontic movement.^9^ ^,^ ^13^ ^,^ ^14^ Hence, detailed orthodontic diagnosis ensures accurate treatment planning and long-term maintenance of periodontal health.^14^ ^,^ ^17^

Treatment planning decisions should be made based on the patient’s documentation. However, there is limited information focusing on the importance of CBCT images in the decision-making process among specialists.^18^ To the best of our knowledge, no previous study has evaluated the importance of CBCT scanning in the diagnosis of BC for orthodontic treatment planning. Thus, the present study aimed to evaluate the impact of alveolar bone coverage assessed through CBCT images on treatment planning by Brazilian orthodontists. The hypothesis was that the CBCT information regarding anterior BC would influence the treatment plan formulated by Brazilian orthodontists.

## MATERIAL AND METHODS

### STUDY DESIGN

An observational and descriptive study, blinded to patient factors, was designed to compare orthodontic treatment plans for malocclusions with critical anterior bone coverage (BC) conditions, based on either 2D information alone or complemmented with additional 3D radiographic data. Treatment plans were simulated using two sequential questionnaires, with the second questionnaire sent immediately after receiving the response for the first. Panoramic radiographs and CBCT images of anterior teeth were used. The study was approved by the Ethical Committee of Federal University of Bahia/Brazil (CAAE: 26024913.0.0000.5024), and involved Brazilian orthodontists recruited from September 2017 to September 2019 via Google Forms questionnaires. Informed consent was obtained from patients to use their photographs and radiographs.

Based on the total number of 31.194 registered orthodontic specialists in the Federal Council of Dentistry in 2017, a representative sample of 38 was computed. Two hundred specialists were invited via email, of whom 159 accepted the invitation and were included in the study.

### CASE SELECTION

Critical bone coverage was defined as bone thickness of 1 mm or less measured in tomographic axial and sagittal sections. Six patient records, four with critical bone coverage detected in CBCT images and two with adequate bone coverage, were selected for the study. The six selected cases were carefully chosen to provide a diversity of clinical situations. Among the cases included, there were two men and four women, with a mean age of approximately 20.83 years and a standard deviation of 5.93 years. CBCT images and reports were included to simulate clinical scenarios. Five cases were from patients without prior orthodontic treatment, and one was a retreatment case. Informed consent was obtained from all patients.

### QUESTIONNAIRES FORMULATION AND APPLICATION

**Figure 1:**
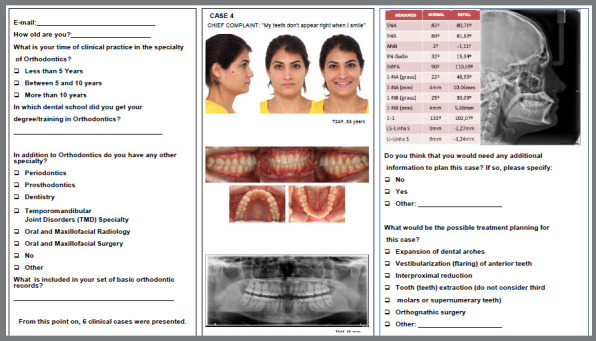
First questionnaire and records of clinical case 4.

**Figure 2:**
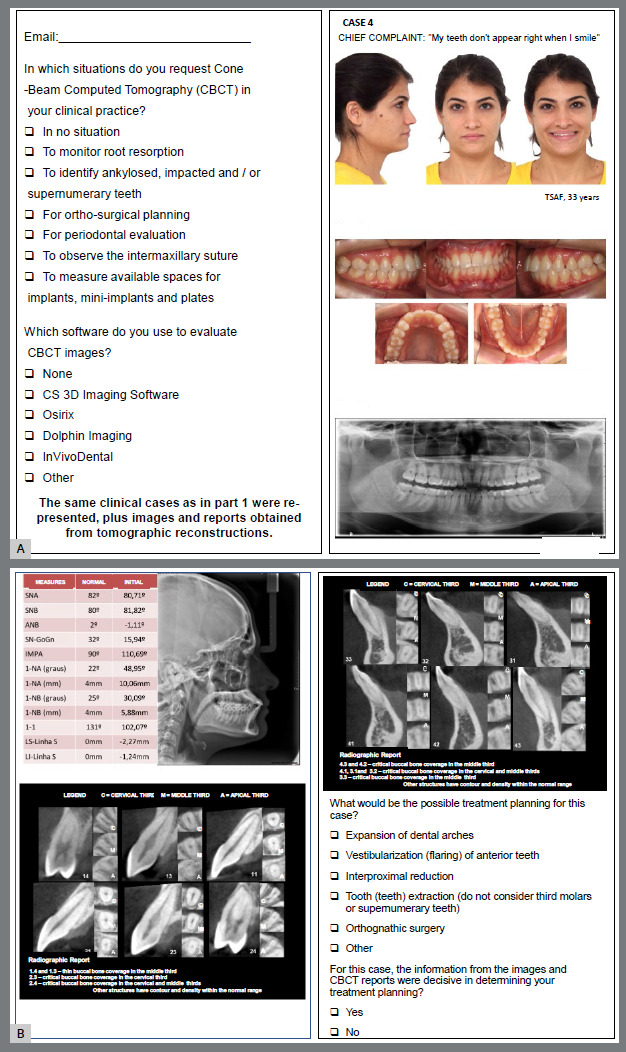
Second questionnaire including extraoral and intraoral photographs, with the patient’s chief complaint, a panoramic radiograph **(**A), a lateral cephalometric radiograph with Steiner’s and Tweed’s analyses, and upper and lower anterior CBCT sagittal images, with a report of incisor bone coverage provided by a radiologist of case 4 **(**B).

Two questionnaires were formulated using the Google Forms platform. The first questionnaire presented the selected cases randomly, each containing extraoral and intraoral photographs, panoramic and lateral cephalometric radiographs. Orthodontists were asked to choose a treatment plan for each case. The provided options included the expansion of dental arches, vestibularization (flaring) of the anterior teeth, interproximal reduction, extraction of tooth/teeth (excluding third molars or supernumerary teeth), orthognathic surgery, and others. These were multiple-choice answers (Fig 1). The second questionnaire included the same cases, with additional slides displaying CBCT images and radiologist reports on bone coverage. The options for answers were the same as those in the first questionnaire. Orthodontists were also asked about the criteria for requesting CBCT in their practice (Fig 2).

### STATISTICAL ANALYSIS

Data from 159 orthodontists who responded to both questionnaires were analyzed descriptively. Participants’ characteristics and CBCT indications were assessed using frequencies. Orthodontists were divided into two groups based on experience, and a chi-square test was used to compare treatment plan changes with and without CBCT information. The McNemar test evaluated differences between the first and second treatment plans proposed by each orthodontist. Statistical analysis was conducted using SPSS version 19.0.

## RESULTS

Among the 159 included participants (mean age ± standard deviation = 42.91 ± 9.47 years, 51% male), most had more than ten years of experience in orthodontic practice (66.7%) and 72.3% had a graduate degree only in orthodontics. The majority of orthodontists who participated in the survey completed their postgraduate studies in orthodontics in the Southeast (51.6%) or in the Northeast (39.6%) of the country. Additionally, they did not advise CBCT routinely (95.6%) for diagnostic purposes. The indications for CBCT used by orthodontists were tooth ankylosis, followed by surgery, tooth impaction, root resorption, periodontal evaluations, and determination of the amount of opening in the intermaxillary suture (Table 1).

**Table 1:** CBCT indication criteria used by the orthodontists (n=159).

CBCT indication	Root resorption n (%)	Tooth ankylosis n (%)	Surgery n (%)	Periodontal investigation n (%)	Intermaxillary suture investigation n (%)	Tooth impaction n (%)
No	109 (68.6)	12 (7.5)	61 (38.4)	128 (80.5)	135 (84.9)	95 (59.7)
Yes	50 (31.4)	147 (92.5)	98 (61.6)	31 (19.5)	24 (15.1)	64 (40.3)

There was a significant statistical difference between the proposed treatment plans in questionnaires 1 and 2. In all six cases, most orthodontists changed the treatment plan after assessing the CBCT images and the radiologist’s report presented in the second questionnaire, as seen in 93.7% (*p*<0.001) in case 5, 78.6% (*p*<0.001) in case 4, 74.2% (*p*<0.001) in case 3, 69.8% (*p*<0.001) in case 6, 66% (*p*<0.001) in case 2 and 61% (*p*=0.01) in case 1. Cases 1 and 3 showed adequate BC in the anterior teeth. Differently, cases 2, 4, 5, and 6 displayed critical BC. Although the participants were divided into two groups according to their experience (G1 with <10 years and G2 with >10 years), both groups showed a statistically significant difference in the proposed treatment plans (Table 2).

**Table 2:** Bivariate analysis of the orthodontic treatment plan before and after CBCT information (n=159).

	Treatment plan, n (%)			
	Changed	Maintained	(95% CI)	p value
Case 1				
Before and after CBCT	97 (61)	62 (39)	(0.53-1.87)	0.01*
Case 2				
Before and after CBCT	105 (66)	54 (34)	(8.38-23.43)	<0.001*
Case 3				
Before and after CBCT	118 (74.2)	41 (25.8)	(16.76-31.29)	<0.001*
Case 4				
Before and after CBCT	125 (78.6)	34 (21.4)	(21.32-35.48)	<0.001*
Case 5				
Before and after CBCT	149 (93.7)	10 (6.3)	(37.37-49.59)	<0.001*
Case 6				
Before and after CBCT	111 (69.8)	48 (30.2)	(12.23-27.07)	<0.001*

McNemar Chi-square test. CI = confidence interval; * *p* ≤ 0.05.

In case 1, 43.4% of orthodontists proposed anterior tooth vestibularization in questionnaire 1, while in questionnaire 2, 36.5% proposed interproximal reduction. In cases 2, 4, 5 and 6, orthognathic surgery was the most reported treatment plan for both questionnaires (<41.5%). In case 3, tooth extraction was the most reported treatment plan in both questionnaires 1 (52.2%) and 2 (55.3%) (Table 3).

**Table 3:** Analysis of the orthodontic treatment plan, n (%), for each case, before (Q1) and after (Q2) CBCT information.

	Case 1		Case 2		Case 3		Case 4		Case 5		Case 6	
TREATMENT PLANS	Q1	Q2	Q1	Q2	Q1	Q2	Q1	Q2	Q1	Q2	Q1	Q2
Expansion of dental arches	18.9^a^	15.1^a^	31.4^a^	22.6^a^	15.7^a^	10.1^a^	5.7^a^	2.5^a^	2.5^a^	1.9^a^	3.1^a^	2.5^a^
Vestibularization (flaring) of anterior tooth	43.4^a^	22.6^b^	10.7^a^	6.3^b^	3.8^a^	1.3^a^	0.6^a^	0.0^a^	0.6^a^	0.0^a^	6.3^a^	7.5^a^
Interproximal reduction	10.1^a^	36.5^b^	3.1^a^	15.1^b^	25.8^a^	28.3^a^	16.4^a^	16.4^a^	-	-	13.2^a^	11.3^a^
Tooth extraction	0.0^a^	2.5^b^	0.6^a^	3.8^a^	52.2^a^	55.3^a^	15.7^a^	16.4^a^	5.0^a^	3.8^a^	7.5^a^	9.4^a^
Orthognathic surgery	0.0^a^	0.6^a^	41.5^a^	42.1^a^	0.6^a^	1.9^a^	52.8^a^	57.9^a^	91.2^a^	94.3^a^	49.1^a^	54.7^a^
Leveling and alignment	27.0^a^	21.4^a^	6.9^a^	4.4^a^	0.6^a^	1.3^a^	5.7^a^	5.0^a^	0.6^a^	0.0^a^	12.6^a^	7.5^a^
Tooth distalization	0.6^a^	0.6^a^	5.0^a^	5.0^a^	0.6^a^	1.3^a^	2.5^a^	1.9^a^	-	-	7.5^a^	5.7^a^
Other	0.0^a^	0.6^a^	0.6^a^	0.6^a^	0.6^a^	0.6^a^	0.6^a^	0.0^a^	-	-	0.6^a^	1.3^a^
*p*-value	>0.001		0.004		0.543		0.720		0.642		0.767	

Each superscript letter denotes a subset of the variable “treatment plan”. In each case, the values with different superscript letters in a line are significantly different (*p*<0.05).

Most participants dismissed the need for CBCT information on case planning in questionnaire 1 in all six cases. In case 6, most orthodontists declared that CBCT information was decisive in treatment planning (Table 4).

**Table 4:** Frequency table of the orthodontist perception of the CBCT importance for the treatment plan, n (%).

	Case 1	Case 2	Case 3	Case 4	Case 5	Case 6
Do you think you would need any additional information to plan this case? *						
No	130 (81.8%)	84 (52.8%)	102 (64.2%)	114 (71.7%)	102 (64.2%)	109 (68.6%)
Model discrepancy or information from 2D images	28 (17.6%)	53 (33.3%)	22 (13.8%)	31 (19.5%)	35 (22.0%)	34 (21.4%)
Information from CBCT image	1 (0.6%)	22 (13.8%)	35 (22.0%)	14 (8.8%)	22 (13.8%)	16 (10.1%)
For this case, the information from the images and CBCT reports were decisive in determining your treatment planning? **						
No	94 (59.1%)	90 (56.6%)	87 (54.7%)	83 (52.2%)	85 (53.5%)	72 (45.3%)
Yes	65 (40.9%)	69 (43.4%)	72 (45.3%)	76 (47.8%)	74 (46.5%)	87 (54.7%)

*Questionnaire 1. **Questionnaire 2.

## DISCUSSION

Data from this study revealed that CBCT information regarding BC of anterior teeth significantly impacted the treatment plans proposed by Brazilian orthodontists. Our findings demonstrated that CBCT examinations lead to a treatment plan different than initially proposed by orthodontists. 

Herein, most participants completed their postgraduate program from the southeastern and northeastern, where accounts the largest number of orthodontic programs in Brazil. Most orthodontists (66.7%) had more than 10 years of experience, which may have influenced the low frequency of CBCT referral (4.4%), since it is a relatively new imaging technique and may imply a previous experience of the examination and software.

Unlike 2D image, CBCT image can be used to measure the thickness and height of buccal alveolar plates with high precision and accuracy.^12^ ^,^ ^13^ ^,^ ^19^ ^,^ ^20^ In other hand, CBCT might overestimate the actual measurements of periodontal defects,^21^ and aspects of image acquisition such as FOV, and voxel size can influence the diagnosis of bone coverage. Nevertheless, voxels smaller than 0.150mm^3^ are reliable for identifying periodontal bone defects.^22^ Thus, CBCT imaging can improve periodontal diagnosis and determine risk assessment before establishing treatment procedures.^10^ Although orthodontists should strive to use a radiation dose “as low as reasonably achievable” (ALARA), CBCT scans of patients with thin dentoalveolar phenotypes and malocclusions requiring tooth movements toward bone boundaries can be invaluable in treatment planning.^10^ The diagnostic accuracy and treatment planning efficacy with CBCT information can change the treatment plan. 

The present study respected ALARA recommendations regarding CBCT indications. The findings indicated that CBCT is requested in cases of tooth ankylosis, surgery, tooth impaction, and root resorption; and, in lower frequencies, while analyzing the periodontal issues and the amount of intermaxillary suture opening. Therefore, in this study it was observed that most orthodontists would overlook the need of CBCT information in the first questionnaire for their treatment plans. 

The frequency of change in treatment plan after CBCT information was substantial in all six cases, despite the years of practice. Clinical decision-making in orthodontic planning is a complex process, and may be based on personal preferences, training, and experience.^18^ Interestingly, significant changes in the treatment plan were observed in all cases. The revelation of significant alveolar defects, such as thin buccal cortical plates, could play an important role in the treatment plan.^18^ ^-^ ^23^ CBCT information can significantly affect the orthodontist’s decision while developing the treatment plan, as observed in this study. It was noticed that orthodontists realized the importance of CBCT imaging while assessing buccal BC, once they understood the technology and were trained to request and interpret the CBCT images.^23^ ^,^ ^24^ ^,^ ^25^

In this study, there were eight treatment plan options and there was variability in the choice of plans among professionals between the two questionnaires. However, there was consistency in the treatment plan when evaluating the choice individually for each case, after the tomographic information. Generally, most changes in planning after CBCT information were to avoid tooth projection, preserving the buccal bone plate, even when the bone coverage was not considered critical.

Treatment of malocclusions requires information regarding the alveolar bone conditions, especially in cases of lack of space management, and the buccolingual direction determination of tooth movement is needed. Conservative approaches, e.g., buccolingual inclination or translation, expansions, and interproximal reductions, can be substituted by extractions or orthognathic surgeries. If adequate knowledge of BC conditions is provided through CBCT information, favorable prognostic can be obtained.^15^ In some cases, it might be advisable to consider soft tissue and/or bone augmentation to prevent deleterious sequelae after orthodontic treatment.^26^ Thus, the present study analyzed the impact of CBCT information on orthodontic treatment planning of cases with thin or critical anterior BC by Brazilian orthodontists, and highlighted its importance. To the best of our knowledge, this is the first study to analyze the effect of BC assessed through CBCT on orthodontist’s decision-making ability. 

This study has strengths and limitations. The main strength was that participants had no previous knowledge about the objective of the research, in a prospective design and with a large sample size included. A major concern was the limited consistency in the orthodontist’s treatment plans; therefore, a greater sample than the calculated number was included.^27^ Furthermore, the second questionnaire was sent immediately after receiving the response to the first, assuring that the orthodontist would have the previous treatment plan in recent memory, avoiding a recall bias. One limitation of this study was that the participants had no chance to examine the patient. Nevertheless, certified orthodontists were trained in defining treatment plans using patient records. Furthermore, the patient’s chief complaint was described for each case. 

The evaluation of BC through CBCT images affected orthodontic diagnosis and treatment planning of Brazilian orthodontists. It constitutes an important tool for diagnostic evaluation, and information obtained through CBCT may change the treatment plan, to avoid creating or augmenting favorable conditions for fenestrations and/or dehiscence. Despite the clinical relevance of the results of this study, the potential effects of the radiation dose of CBCT can not be underestimate.^28^ ^-^ ^30^ Therefore, the orthodontist should weigh the potential risks and benefits in diagnosis and treatment planning.

## CONCLUSION

In conclusion, significant differences in orthodontic treatment planning using 2D and 3D information were observed, regardless of the professional experience. Information obtained through CBCT regarding alveolar BC influenced the treatment plan; therefore, in selected cases of borderline malocclusion or thin periodontal profile, CBCT can be a valuable tool for orthodontic diagnosis and treatment planning.
